# Genito-Urinary and Syphilitic Diseases

**Published:** 1899-09

**Authors:** Byron H. Daggett

**Affiliations:** Buffalo, N. Y.


### GENITO-URINARY AND SYPHILITIC DISEASES.

CONDUCTED BY BYRON H. DAGGETT, M. D., Buffalo, N. Y.

### GONORRHEAL INFECTION.

COLOMBINI (*Cent. f. Bakteriologie*; December 30, 1898; *Albany Med. Annals*, April, 1899,) reports an unusual case of gonorrheal infection. The patient, a young man, had an attack of gonorrhea, followed by the formation of an abscess in the groin, epididymitis and parotid gland. The urine contained albumin, casts and blood. The suppuration gradually ceased and the urine cleared.

The gonococcus, presenting the usual morphological and cultured properties, was found in the discharges from all the suppurating glands, it also reproduced its kind by inoculation into a healthy urethra.

---

### SYPHILIS, INSANITIES AND PSEUDO-INSANITIES: PROGNOSIS AND TREATMENT.

Chas. K. Mills, before the New York Academy of Medicine, March 16, 1899. In nervous syphilis there are three classes of diseased processes to be taken into consideration—namely, (1) True syphilitic lesions, due to the continuing action of the specific virus; (2) secondary softenings, indurations and degenerations; (3) Para or metasyphilitic affections, degenerative diseases, remote consequences of the virus.

These parasyphilitic diseases are the issue of syphilis; they are "products of its action, under its influence, and without it in all probability would not be present: but although thus proceeding from syphilis, are not of a syphilitic (specific) nature." —Mickle.

If there be a clear diagnosis of specific nondegenerative lesions, without marked secondary changes, the prognosis under specific medication is good.

The larger majority of cases present the combination of three specific lesions and secondary degenerations and parasyphilitic disease. The true specific lesions may be controlled or influenced by special medication. The most important forms of syphilitic insanity are as follows: (1) Syphilitic mental disorders due to the presence in the blood of the specific toxin (syphilemia) without the signs of degenerative disease being present; (2) dementia paralytica or general paralysis, uncomplicated by parasyphilitic disease; (3) syphilitic pseudoparesis.

True dementia paralytica is a parasyphilitic, progressive, degenerative disease. The first division includes the syphilitic toxemic insanities, such as syphilitic melancholia, mania and syphilitic acute dementia. Profound syphilitic melancholia may be preceded by neurasthenia, physical and mental exhaustion, most marked in the secondary stage and should not be confounded with disorders of nutrition or psychic disturbances, due to apprehension and remorse.

Syphilitic melancholia is the manifestation of a specific toxemia. Syphilitic melancholia is characterised by depression, depressive hallucinations, by hypochondrial delusions and delusions of suspicion, persecution and poisoning, often with suicidal tendencies. In syphilitic mania there are often rapidly changing hallucinations and delusions, with incoherence, also associated with mental exaltation, varying from hypomania to delirious mania. In other cases the leutic virus may cause stupor, torpidity and apathy in various degrees. Syphilitic insanity may exist without presenting specific lesions or degeneration of the noble elements of the nervous system. Insanities and pseudo-insanities, due to syphilitic toxemias, without recognisable organic lesions, under early and active treatment, warrant a favorable prognosis.

Incurable dementia and chronic mania will not be the termination of this stage in the hands of a skilful therapeutist. The mercurial or the mixed treatment is the most applicable. Mercury should be administered for its constitutional effects and the iodides in increasing doses.

Tabes and paralytic dementia are practically the same disease, syphilitic pseudotabes and pseudoparesis are comparable affections. True uncomplicated tabes and general paralysis are parasyphilitic diseases. 80 to 90 per cent. of all general paretic cases are syphilitic. A syphilitic toxin may dwell in the blood for years and years inert and inocuous, or under morbific activity it may set up disease in any of the tissues of the body. Syphilitic pseudoparesis is far more common than true pseudotabes and its lesions are protean and numerous. The prognosis and treatment of syphilis must be based upon a thorough differential diagnosis and the treatment guided by an investigation of its various forms, including the history of the treatment pursued and the idiosyncrasies of the case. For prognosis and treatment there must be a clear distinction made between specific lesions and parasyphilitic or metasyphilitic affections. The curable syphilitic diseases bear recent specific lesions. According to the degree of involvement of the true nerve elements is prognosis unfavorable and treatment unavailing. In no branch of medicine and surgery is accurate and thorough diagnostic study of cases more important than in the treatment of nervous syphilis. The physician should be competent to diagnosticate and treat syphilitic disease without making experimental therapeutic test. The mixed treatment is the most valuable for the chronic forms of nervous syphilis.

### THE USE OF THE URINE SEGREGATOR IN THE DIAGNOSIS OF DISEASES OF THE URINARY TRACT.

Dr. Harris, of Chicago, before the New York Academy of Medicine (*Journal of Cutaneous and Genito-urinary Diseases*, May, 1899). The purpose of the instrument (Harris segregator) is the separate collection of the urine from each kidney. This instrument, by means of a lever in the rectum or vagina, raises a septum in the bladder. A double catheter, or rather two catheters with Condé beaks are introduced into the bladder through a common shield and the catheters are rotated so that the beaks dip over the septum, one on each side of the bladder. In this way the urine from each ureter is taken up and carried out to separate vials. The technique of the use of the instrument is not difficult and the errors or failures reported are the result of faulty manipulation. The patient should lie flat upon a table with thighs flexed.

The bladder should be irrigated with sterilised water through the segregator. The instrument should be adjusted while the bladder is distended with the sterilised fluid by blocking the return flow. The catheter must be in the median line while the lever is inserted and fixed. The lever should be kept in a horizontal line. The catheters are then rotated to their fullest extent and held by small spiral spring and the large spiral spring is caught into one of the notches of the lever with tension sufficient to produce moderate pressure. The instrument is gently withdrawn until resistance is felt. The fluid is then allowed to escape from the bladder and the vials and hand bulb are attached. The hand bulb should be gently used until the flow of urine is started. The flow is intermittent and regular and comes in drops. If the flow comes from each side we may be certain of the existence of two kidneys. The location of pathological process in the urinary system may be determined by this instrument. The instrument should be left in place for thirty minutes and can be used without anesthetics in the majority of cases.

The contra indications for the use of this instrument are distorted bladders, vesicle calculi and excessively enlarged prostates.

Dr. Sondern said that this instrument was satisfactory in its results in most cases, but it was inferior or less reliable than the methods employed by Casper and Kelly by direct catherisation of the ureters, but its technique was less difficult. The Harris instrument, like all others, had its limitations.

Dr. Elsberg expressed admiration for this ingenious instrument. The speaker reported various experiments with the use of the Harris segregator, and said that it could be used by any one, so that examination of the separated urines would become more general and thereby our knowledge of renal disease advanced.

---

SOME OBSERVATIONS ON THE PROSTATE.

R. H. Greene and J. W. Blanchard (*Journal of Cutaneous and Genito-urinary Diseases*, January, 1899,) report the results found in the examination of 214 cases of urethritis which presented a discharge from the anterior urethra. The examinations were made to determine the relative frequency of the involvement of the posterior urethra. The prostate was examined in all cases. The prostatic secretion was examined microscopically in twenty-nine cases. The anterior urethra was first washed, the urine was then passed and examined for pus. These cases presented no indications of bladder involvement. The prostate was examined by the finger passed into the rectum. Secretion from the prostate was obtained by digital massage and examined microscopically. In some cases the secretion was extremely scanty, but in a large majority of cases an amount consisting apparently of several drops could be seen in the urine passed. Of 214 cases all gave a history of one or more attacks of gonorrhea, except four. The posterior urethra was found involved in 142 cases—66 per cent.—and the anterior in 72 cases or 34 per cent. The examination of the prostate showed an enlargement to the finger in 102 cases or 47 per cent. of all. The enlargement or swelling was marked in the left lobe of the prostate in 71 per cent. of these cases; and 19 per cent. in the right lobe. General enlargement in 10 per cent. of the cases in which any enlargement was present.

Dr. Blanchard reports examination of twenty cases and the following is an example of his findings

Case 158.—1, pus; 2, leucocytes; 3, few eosinophiles; 4, very many small lymphocytes; 5, very many erythrocytes; 6, large and small spheroidal epithelium, with eccentric nucleus, few with central nucleus; 7, few bodies without granules, few bacteria.

Out of twenty specimens there were pus in sixteen and diplocci in three. The most ordinary microscopic presentations were pus, mucus, and corpora amylacea, indicating infiltration into the prostatic follicles. The presence of lymphocytes would probably indicate infiltration or some change in the interstitial tissue. There are two forms of posterior urethritis. One apparently confined to the surface the other dipping down into the prostate. Congestive inflammation of the prostate frequently accompanies urethritis, which does not cause abscess formation. Whether the prostate is fully restored to its normal condition is unknown. We also find enlargements not due to urethritis. There are many mysteries connected with this organ. The evidence of those who have studied urethritis in the male, with the exception of the reports of a few gentlemen, who allege that it is an ephemeral disease, which can be cured by a few irrigations, tend to show that as in the female it is a serious affection and that it often involves structures remote from its infecting nidus, and this may occur at any time during the existence of the disease.

